# Promising Perceptions, Divergent Practices and Barriers to Integrated Malaria Prevention in Wakiso District, Uganda: A Mixed Methods Study

**DOI:** 10.1371/journal.pone.0122699

**Published:** 2015-04-02

**Authors:** David Musoke, George Miiro, George Karani, Keith Morris, Simon Kasasa, Rawlance Ndejjo, Jessica Nakiyingi-Miiro, David Guwatudde, Miph Boses Musoke

**Affiliations:** 1 Department of Disease Control and Environmental Health, School of Public Health, Makerere University College of Health Sciences, Kampala, Uganda; 2 Uganda Virus Research Institute, Entebbe, Uganda; 3 Cardiff School of Health Sciences, Cardiff Metropolitan University, Wales, United Kingdom; 4 Department of Epidemiology and Biostatistics, School of Public Health, Makerere University College of Health Sciences, Kampala, Uganda; 5 Medical Research Council, Uganda Virus Research Institute, Entebbe, Uganda; 6 School of Sciences, Nkumba University, Entebbe, Uganda; Centers for Disease Control and Prevention, UNITED STATES

## Abstract

**Background:**

The World Health Organization recommends use of multiple approaches to control malaria. The integrated approach to malaria prevention advocates the use of several malaria prevention methods in a holistic manner. This study assessed perceptions and practices on integrated malaria prevention in Wakiso district, Uganda.

**Methods:**

A clustered cross-sectional survey was conducted among 727 households from 29 villages using both quantitative and qualitative methods. Assessment was done on awareness of various malaria prevention methods, potential for use of the methods in a holistic manner, and reasons for dislike of certain methods. Households were classified as using integrated malaria prevention if they used at least two methods. Logistic regression was used to test for factors associated with the use of integrated malaria prevention while adjusting for clustering within villages.

**Results:**

Participants knew of the various malaria prevention methods in the integrated approach including use of insecticide treated nets (97.5%), removing mosquito breeding sites (89.1%), clearing overgrown vegetation near houses (97.9%), and closing windows and doors early in the evenings (96.4%). If trained, most participants (68.6%) would use all the suggested malaria prevention methods of the integrated approach. Among those who would not use all methods, the main reasons given were there being too many (70.2%) and cost (32.0%). Only 33.0% households were using the integrated approach to prevent malaria. Use of integrated malaria prevention by households was associated with reading newspapers (AOR 0.34; 95% CI 0.22 –0.53) and ownership of a motorcycle/car (AOR 1.75; 95% CI 1.03 – 2.98).

**Conclusion:**

Although knowledge of malaria prevention methods was high and perceptions on the integrated approach promising, practices on integrated malaria prevention was relatively low. The use of the integrated approach can be improved by promoting use of multiple malaria prevention methods through various communication channels such as mass media.

## Introduction

The current core global malaria prevention interventions are long lasting insecticidal nets (LLINs) and indoor residual spraying (IRS) which have demonstrated impact in reducing malaria [1,2]. These malaria prevention methods are being used in several malaria endemic countries including Uganda. Although use of LLINs has been extensively promoted throughout Uganda in recent years including using mass distributions campaigns, IRS implementation has only occurred in selected districts particularly in the northern part of the country. Despite current malaria prevention efforts, the disease still causes a significant burden globally. In 2012, there were an estimated 207 million cases of malaria worldwide, 80% occurring in sub-Saharan Africa [3]. Malaria is the leading cause of morbidity and mortality in Uganda particularly among children under 5 years of age [4]. This disease burden has necessitated use of more innovative strategies to prevent occurrence of malaria.

The integrated approach to malaria prevention is being increasingly explored as a strategy to prevent malaria. The approach advocates for the use of several malaria prevention methods in a holistic approach. The malaria prevention methods in the integrated approach include those that offer individual / household protection (insecticide treated nets (ITNs), insecticide sprays, body mosquito repellents, IRS and mosquito coils); reduce mosquito breeding sites (draining pools of water, larviciding and clearing unnecessary vegetation around homes); and reduce entry of mosquitoes into houses (installing mosquito screening in windows, ventilators and open eaves, and closing windows and doors early in the evenings). These methods have been shown to individually contribute towards reducing mosquito populations and mosquito bites thereby preventing the spread of malaria [5,6,7].

The use of appropriate combinations of non-chemical and chemical methods of malaria vector control in the context of integrated vector management has been recommended by the World Health Organization [8]. Indeed, a combination of malaria prevention strategies has been shown to have greater impact than single methods in several studies [9,10,11]. However, these studies have focused mainly on the use of ITNs and IRS hence little evidence on research involving more than 2 interventions. Integrated malaria prevention has several benefits including providing multiple barriers against mosquito vectors, offering protection for entire households, and also contributing to preventing other vector borne diseases. It is therefore important to explore the integrated approach to malaria prevention as a strategy to complement existing malaria control interventions and approaches.

A recent pilot study using the integrated approach to malaria prevention was well received by rural communities in Uganda [12]. However, the general perceptions on the use of the approach were not assessed, neither has it been investigated in any other studies in Uganda and elsewhere. Before further research is carried out on the integrated approach, it is pertinent that community perceptions on the strategy are assessed. In addition, establishing the extent of use of the approach is necessary so as to provide information on current malaria prevention practices by communities. This study assessed perceptions and practices on integrated malaria prevention in Wakiso district, Uganda.

## Methods

### Ethics statement

Ethical approval to conduct the study was obtained from Makerere University School of Public Health Higher Degrees, Research and Ethics Committee. The research was also approved and registered at the Uganda National Council for Science and Technology. Participation in the study was voluntary and participants provided written informed consent after explaining to them the purpose of the research including the anticipated risks and potential benefits before taking part.

### Study design and data collection

The study was a clustered cross-sectional survey at household level conducted in 2013 involving both quantitative and qualitative data collection methods. The clusters were villages while the study units were households. Heterogeneity between clusters was assumed especially in terms of exposure to malaria. However, exposure within clusters was expected to be similar. Quantitative data were collected using a questionnaire while focus group discussions (FGDs) provided qualitative data. The questionnaire assessed the participants’ knowledge, perceptions, practices, and anticipated challenges on integrated malaria prevention and was administered once for each household involved in the study. The study participants were household heads or other responsible adults (such as spouses). These participants were used as they were expected to be knowledgeable on the malaria prevention strategies used by their households. Six FGDs (3 male, 3 female) were conducted to assess the perceptions on the various malaria prevention methods and use of the integrated approach to prevent the disease. Participants for the FGDs were selected from the general population in 3 randomly selected villages and each FGD comprised of between 8 to 12 participants. Each selected village provided participants for 1 male and 1 female FGD.

### Study area and sampling

The study was carried out in Ssisa sub-county, Wakiso district located in the central region of Uganda. Ssisa sub-county is predominantly a rural area made up of 11 parishes which were all involved in the study. Using the formula for cross-sectional studies [13], and catering for the clustering effect using a cluster size of 20, taking a rate of homogeneity *roh* of 0.025 [14], design effect of 1.5, and non-response rate of 10%, a minimum sample of 644 was required. The cluster size was increased from 20 to 23 so as to maintain the minimum number of clusters at 28. The number of villages from each parish involved in the study was determined as proportionate to the number of households per parish [data obtained from Uganda Bureau of Statistics (UBOS)] which ranged from 1 to 7. For parishes which provided more than 1 village, random sampling was used to identify those involved in the study. Using the village chairperson’s home as the starting point, data collectors systematically selected households to be involved in the study. The household sampling interval, which ranged from 3 to 13, was determined by the number of households in each village as obtained from UBOS.

### Data analysis

Quantitative data were entered in SPSS version 10.0 (Chicago, Illinois, USA) and analysed in STATA version 10.0 (College Station, Texas, USA). Perceptions on the integrated approach were assessed both quantitatively and qualitatively. Quantitatively, participants were asked about their awareness of the various malaria prevention methods in the integrated approach. Awareness was defined as knowledge on the possible use of a method for preventing malaria. Assessment on awareness of malaria prevention methods was done by prompting. These methods were: sleeping under untreated mosquito net, sleeping under ITN, installing screening in windows, installing screening in ventilators and open eaves, removing mosquito breeding sites, spraying house with insecticides, clearing overgrown vegetation around homes, closing windows and doors early in the evenings, larviciding in water pools, indoor residual spraying, using body mosquito repellents, and using mosquito coils. Furthermore, they were asked whether they would use all the methods in the integrated approach in a combined manner if trained. For participants who would not use all the methods, reasons for their preferences were assessed. In addition to establishing the individual methods used to prevent malaria, utilization of integrated malaria prevention was defined as using at least two methods by a household.

To assess the association between socio-demographic variables, malaria prevention associated factors, and integrated malaria prevention, frequencies and associated proportions were obtained for each variable category. The 95% confidence interval (CI) of the proportions were obtained using the analytically derived variance estimator associated with the sample proportion—SE(p) = √{p(1-p)/n}. The association between each variable and the main outcome (integrated malaria prevention) was analysed using logistic regression. Principal component analysis (PCA) was done to select variables to include in the socio-economic status (SES) index. Initially, there were 7 variables considered: source of water; toilet facilities; fuel for cooking; materials of the floor; vehicle ownership; a wealth index composed of ownership of TV, refrigerator, telephone and radio; and having electricity. Vehicle ownership was computed using a score which composed of possession of a bicycle, motorcycle and car. The categories of each of the variables were arranged from worst to best, with the best scoring the highest points. The PCA yielded 5 variables (water source, toilet facilities, cooking fuel, floor material, and wealth index) as the best predictor for SES and these were used to compute the SES index used in the analysis. After ordering the SES index, households were divided into terciles with those scoring lowest considered as the poorest while those with the highest score were considered the richest. The remaining 2 variables (having electricity and vehicle ownership) were analysed individually in the crude analysis. A multivariable model was fitted using multiple logistic regression with robust SEs to account for clustering within villages. All variables with p-values ≤0.1 and the known prior confounders (age and sex) were added into the model by backward stepwise regression. The significance of each additional variable was tested using log likelihood and if not significant, was excluded from the model. The variables initially included in the multivariable model were: age, sex, education level, occupation, family size, whether a household had a child under 5 years, whether there was a pregnant woman in the household, reading newspapers, listening to radio, and vehicle ownership. Variables which remained significant at the end of model fitting made up the final regression model.

Qualitatively, the FGDs established what the participants thought about the integrated approach and explored why some of the methods were likely not to be used. All FGDs were tape recorded and later transcribed verbatim in *Luganda*, the main local language used in central Uganda. They were then translated into English and reviewed several times by the principal investigator and another researcher. By using a qualitative thematic analysis, data were coded into initially predetermined themes and other emerging ones. This was done using QSR International’s NVivo version 10 qualitative data analysis software. The data with developed themes was then examined by an independent investigator to ensure all data had been correctly coded and assigned to appropriate themes. Direct quotations from the FGDs are presented in italics to highlight key findings.

## Results

A total of 727 households were selected from 29 villages with a minimum of 23 households obtained from each village. All households sampled accepted and took part in the study. The FGDs were participated in by a total of 58 individuals, 27 men and 31 women.

### Socio-demographics of participants

The mean age of participants was 32 years, over two thirds (67.8%) were female while a third were involved in business (34.2%) and farming (32.3%). Nearly half of the participants (45.3%) had primary as their highest academic level attended and only 3.2% having reached tertiary/university. Over half of the participants (53.7%) earned less than 40 US dollars per month in their households. Nearly half of the participants (47.7%) had household size between 3 and 5 members while 32.7% had at least two children under 5 years of age in their household (Table 1).

**Table 1:** Socio-demographic characteristics of participants.

Variable	Frequency (n = 727)	Percentage (%)
**Gender**		
Male	234	32.2
Female	493	67.8
**Age (years)**		
18–29	289	39.8
≥ 30	438	60.3
**Religion**		
Catholic	294	40.4
Anglican	220	30.3
Pentecostal	95	13.1
Muslim	94	13.0
Seventh Day Adventists	19	2.6
Others	5	1.0
**Occupation**		
Farmer	235	32.3
Business	249	34.2
Housewife	82	11.3
Unemployed	108	14.9
Others	53	7.3
**Highest level of education**		
None	78	10.7
Primary	329	45.3
Secondary (ordinary) level	259	35.6
Secondary (advanced) level	38	5.2
Tertiary / university	23	3.2
**Average household monthly income (US dollars)**		
< 40	390	53.7
≥ 40	337	46.4
**Position in household in relation to household head**		
Household head	416	57.2
Spouse	227	31.2
Parent	35	4.8
Sibling	20	2.8
Other relative / not related	29	4.0
**Household size (median 5, upper range 15)**		
1–2	112	15.4
3–5	347	47.7
≥ 6	268	36.9
**Number of children under 5 years in household**		
None	254	34.9
1	235	32.3
≥ 2	238	32.7

### Awareness, perceptions and barriers to use of integrated malaria prevention

Participants were highly aware of the various malaria prevention methods in the integrated approach. These included use of ITNs (97.5%), removing mosquito breeding sites (89.1%), clearing overgrown vegetation near houses (97.9%), and closing windows and doors early in the evenings (96.4%). The least known methods were use of body mosquito repellents (48.0%), IRS (59.3%) and larviciding (59.4%) (Table 2).

**Table 2:** Awareness of malaria prevention methods in the integrated approach.

Methods categorised by target	Frequency(n = 727)	Percentage (%)
**Personal protection**		
Sleeping under untreated mosquito net	721	99.2
Sleeping under ITN	709	97.5
Spraying house with insecticides	680	93.5
Using mosquito coils	633	87.1
Indoor residual spraying	431	59.3
Using body mosquito repellents	349	48.0
**Mosquito breeding**		
Clearing overgrown vegetation around homes	712	97.9
Removing mosquito breeding sites	648	89.1
Larviciding in water pools	432	59.4
**Entry of mosquitoes into houses**		
Closing windows and doors early in the evenings	701	96.4
Installing screening in ventilators and open eaves	576	79.2
Installing screening in windows	569	78.3

When participants were asked if their households would use all the suggested malaria prevention methods (in Table 2 above) in a combined manner to prevent malaria if trained, over two thirds (68.6%) said they would. Among those who said their households would not use all the methods (n = 228), the main reasons given included their being too many hence would use only some of them (70.2%, n = 160) and very costly (32.0%, n = 73). The main methods that would not be used were body mosquito repellents (81.1%, n = 185), larviciding (63.2%, n = 144), IRS (62.3%, n = 142) and mosquito coils (53.1%, n = 121) (Table 3).

**Table 3:** Malaria prevention methods that households would not use in the integrated approach.

Methods categorised by target	Frequencies (n = 228)	Percentage (%)
**Personal protection**		
Sleeping under untreated mosquito net	18	7.9
Sleeping under ITN	83	36.4
Spraying house with insecticides	83	36.4
Using mosquito coils	121	53.1
Indoor residual spraying	142	62.3
Using body mosquito repellents	185	81.1
**Mosquito breeding**		
Clearing overgrown vegetation around homes	45	19.7
Removing mosquito breeding sites	74	32.5
Larviciding in water pools	144	63.2
**Entry of mosquitoes into houses**		
Closing windows and doors early in the evenings	29	12.7
Installing screening in ventilators and open eaves	83	36.4
Installing screening in windows	88	38.6

From the FGDs, participants generally supported the use of several methods in a holistic approach to prevent malaria in their households. However, they were concerned about the cost involved in implementing some methods such as ITNs, installing screening in windows and ventilators, larviciding, spraying insecticides and use of mosquito repellents.

*“Owning mosquito nets wouldn’t be a problem but people do not have the means of buying them*. *That is why if they have any slight hope that the Government is to provide them with nets*, *they would wait for them not knowing that this could delay and they may even fall sick as they still wait for those free nets*.*”* Male FGD participant.

*“We have seen insecticides in shops but they are not used because we cannot afford to buy them*. *In this village*, *you may find that there is only one person who uses DOOM*. *In fact*, *I can’t even find a single can of DOOM* *doom in my house*.*”* Male FGD participant.

*“The use of larvicides in ponds of water is a good option to prevent malaria in our village but they are too expensive*. *If one tries to calculate the amount of larvicides required for the numerous water ponds resulting from brick making we have in this area*, *it is almost impossible to afford*.*”* Female FGD participant.

Lack of knowledge about some of the methods in the integrated approach was also identified as a limitation in using them. This included practices which were known but not thought to contribute towards malaria prevention such as installing screens in windows and ventilators, and methods which had not been heard at all by some participants notably skin repellents.

*“I did not know that putting screening in windows was important to prevent mosquito entry into houses*. *We are actually not familiar with this malaria prevention method*. *All we do is only install iron bars in our windows to prevent thieves from entering our houses*. *I am therefore learning this for the first time*.*”* Male FGD participant.

*“We do not know about use of substances smeared on our skin* [repellents] *to chase away mosquitoes*. *Can these substances be available*? *Many of us do not know about those things unless you bring us some samples*. *However*, *don’t those substances have any side effects when used by people*?*”* Female FGD participant.

There were also concerns about the potential side effects of some methods to health hence being reluctant to use them. The methods that were feared to have health effects when used were IRS, body repellents, mosquito coils and spraying houses with insecticides.

*“We were told that mosquito coils have side effects on our health and that the smoke from them can be harmful*. *They said it requires smoldering them before entering the house but some of us may enter when the coil is still burning*. *Those coils may just not work for us*.*”* Female FGD participant.

*“Isn’t that chemical used to spray on the walls* [IRS] *harmful to our health and wellbeing*? *In our homes*, *we have children who like playing and they may touch sprayed walls and then hold whatever they are eating*. *Wouldn’t those chemicals affect them when ingested*?*”* Female FGD participant.

*“Besides insecticides being expensive*, *some people say they could be harmful to our health just like DDT* [Dichloro Diphenyl Trichloroethane]. *Isn’t that true*?*”* Female FGD participant.

### Practices on integrated malaria prevention

**Fig 1:**
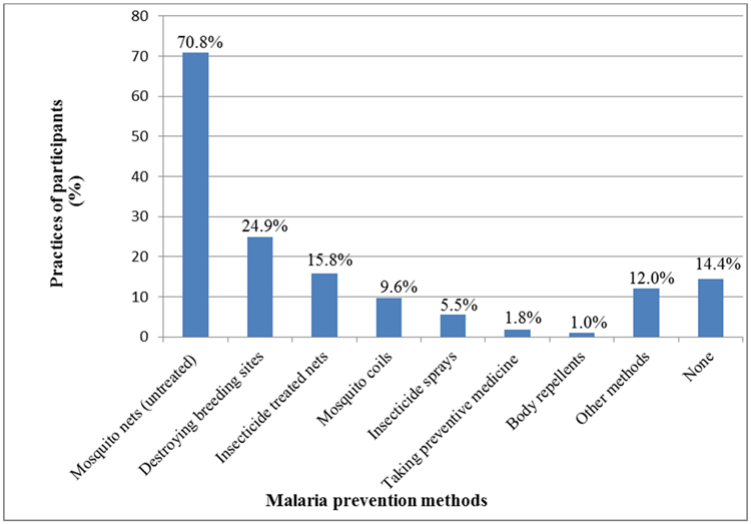
Malaria prevention methods used by households.

Over two thirds of participants (70.8%, n = 515) used untreated mosquito nets to prevent malaria in their households while ITNs were used by 15.8% (n = 115) of households. Other methods in the integrated approach such as destroying mosquito sites (24.9%, n = 181) and use of body repellents (1.0%, n = 7) were used by few households. Among the participants, 14.4% (n = 105) said their households were not using any method to prevent the occurrence of malaria (Fig 1).

Among the households, 33.0% were using integrated malaria prevention. Use of integrated malaria prevention at households was associated with reading newspapers (AOR 0.34; 95% CI 0.22–0.53) and ownership of a motorcycle/car (AOR 1.75; 95% CI 1.03–2.98). Households with a family size of 3–5 members were less likely to use integrated malaria prevention (AOR 0.55; 95% CI 0.37–0.83) (Table 4). Variables that were not associated with integrated malaria prevention in the crude analysis were: age, religion, household income, watching TV, having electricity in the house, and socio-economic status.

**Table 4:** Crude and multivariable analysis for use of integrated malaria prevention at households.

Variables	Use integrated malaria prevention		Crude analysis	Multivariable analysis^1^	
	**Frequency**	**% [95%CI]**	**Odds ratio [95% CI]** ^2^	**Adjusted odds ratio [95% CI]** ^2^	**P-value**
**All households**	240	33.0 [29.6–36.4]			
**Sex**					
Male	92	39.3 [33.2–45.7]	Ref	Ref	
Female	148	30.0 [26.1–34.2]	0.66 [0.48–0.92]	1.00 [0.69–1.46]	1.00
**Age (years)**					
18–29	86	29.8 [24.8–35.3]	Ref	Ref	
≥30	154	35.3 [30.8–39.8]	1.28 [0.93–1.76]	1.25 [0.88–1.79]	0.20
**Family size**					
1–2	48	42.9 [34.0–52.2]	Ref	Ref	
3–5	98	28.2 [23.7–33.2]	0.52 [0.34–0.82]	0.55 [0.37–0.83]	0.004*
≥6^3^	94	35.1 [29.6–41.0]	0.72 [0.46–1.13]	0.61 [0.33–1.12]	0.11
**Pregnant woman in household**					
None	209	34.5 [30.8–38.4]	Ref	Ref	
Yes^4^	31	25.6 [18.6–34.2]	0.65 [0.42–1.02]	0.62 [0.34–1.13]	0.12
**Reading newspapers**					
Yes	130	49.8 [43.7–55.9]	Ref	Ref	
No	110	23.6 [20.0–27.7]	0.31 [0.23–0.43]	0.34 [0.22–0.53]	<0.0001*
**Vehicle ownership**					
None	116	27.5 [23.4–32.0]	Ref	Ref	
Bicycle^5^	57	38.8 [31.2–46.9]	1.67 [1.13–2.48]	1.51 [0.88–2.60]	0.13
Motorcycle/car	67	42.4 [34.9–50.2]	1.94 [1.33–2.84]	1.75 [1.03–2.98]	0.04*

* p-value <0.05

^1^ Multivariable analysis included all the 727 households

^2^ 95% CI estimated using robust SE.

^3^ Without account for clustering, 95% CI = 0.37–1.00, p-value = 0.05

^4^ Without account for clustering, 95% CI = 0.39–0.98, p-value = 0.04

^5^ Without account for clustering, 95% CI = 0.99–2.30, p-value = 0.05

From the FGDs, only some of the methods in the integrated approach were used for prevention of malaria. The most used methods were sleeping under mosquito nets, removing potential mosquito breeding sites and clearing overgrown vegetation near houses. The community had previously received ITNs from the Government mainly for children under 5 years of age and pregnant women. However, it was noted that some of the nets were not used for the intended purpose. For instance, some people sold them while those that were initially used had since become old hence discarded.

*“Several people used to sleep under mosquito nets provided to them by the Government many years ago to prevent getting malaria*. *These nets have now become old hence the need to buy new ones which requires resources or hope that the Government can give us new ones very soon as promised*.*”* Female FGD participant.

*“The most common malaria prevention method used in this village is sleeping under mosquito nets but this method is not widespread and there are individuals who got nets from Government and have not used them*. *I have evidence on this as some of the people sold the nets they received to buy alcohol*. *So regarding prevention of malaria*, *we need to think of other ways we can fight the mosquitoes that transmit the disease*.*”* Male FGD participant.

*“Staying near water ponds also leads to being close to the mosquitoes and this would lead to being bitten frequently especially when there are also some bushes around*. *To avoid this*, *we drain all water from water ponds around our homes and slash the bushes*. *This ensures that the mosquitoes are kept away from us and we can then be able to prevent malaria*.*”* Male FGD participant.

## Discussion

The study established that there was very high awareness of most malaria prevention methods that are part of the integrated approach. There was also high willingness to use the integrated approach when trained on the various methods. However, the main reasons for dislike of the integrated approach were methods being too many and costly. Poverty has been shown to affect malaria prevention practices in several studies including use of ITNs [15,16]. Since many communities in Uganda have faced challenges in using single malaria prevention methods due to cost, it could be a barrier to using the integrated approach in which some of the methods require finances. However, cost should not be a hindrance to applying some of the practices in the integrated approach such as early closing of windows and doors, and removing mosquito breeding sites. It is therefore important that communities implement measures in the integrated approach that they have the capacity to do so. In addition, vertical malaria prevention programmes such as free distribution of LLINs by Government is likely to support household and community initiatives in the use of integrated malaria prevention. Although it may not be feasible for every household to use all methods in the suggested approach, it is evident that integrated vector control contributes to greater reductions in malaria transmission than single methods [17]. Indeed, integrated malaria interventions led to control of malaria in Zambia [18] and elimination of mosquito species from Brazil [19] and Egypt [20]. It is therefore prudent to continue promoting integrated malaria prevention in ongoing and future malaria control programmes in Uganda and other endemic countries.

The most unpopular methods of malaria prevention in the integrated approach were mosquito repellents, larviciding, IRS and mosquito coils. There were concerns expressed by participants on potential effects on human health through the use of these methods as previously reported [21,22] which can affect their use in integrated malaria prevention. Adequate information on the various methods is therefore crucial to promote use of multiple methods. Lack of knowledge on some methods, such as screening of windows and ventilators is an obstacle to using the integrated approach being investigated. Indeed, inadequate knowledge on malaria prevention methods negatively affects practices in communities [23,24]. Promotion of the various methods in the integrated approach through sensitization is therefore likely to improve community practices hence reduction in occurrence of malaria.

The most used method in the integrated approach to prevent malaria was mosquito nets (untreated—70.8%; ITNs—15.8%). Some of the ITNs could have been reported as untreated as LLINs (including those provided to the community by Government) are pre-treated hence users do not have to treat them which could have led to the misclassification. This widespread use of mosquito nets can be attributed to national campaigns including net distribution and publicity in Uganda [25] as is the case in other malaria endemic countries. However, the low use of other methods by households is an indication that they are given lower priority for malaria prevention. As the methods in the integrated approach have been shown to individually contribute towards malaria prevention [5,6,7,26], it is necessary that malaria control programmes include them in their packages.

The use of integrated malaria prevention in households involved in this study was low, with only 33.0% using two or more methods to prevent malaria. Indeed, the use of other malaria prevention methods beyond ITNs has been found to be limited in Uganda and elsewhere [27,28]. Given the several methods in the integrated approach available for use by communities, it is ideal that households use multiple methods to prevent malaria as opposed to single ones. Reading newspapers was associated with a 3-fold increase in the use of integrated malaria prevention (AOR 0.34; 95% CI 0.22–0.53). This finding emphasises the importance of information as a key factor in promoting health practices including on malaria prevention. Indeed, it has been found in several studies that increased knowledge through access to information is associated with desirable malaria prevention practices [29,30,31]. Although these studies were assessing practices on individual malaria prevention methods notably ITNs, it is likely to be similar to use of multiple methods. To increase use of the integrated approach to prevent malaria, it is important that the ministries of health and other stakeholders in malaria control such health workers sensitize the public about the several malaria prevention methods through various communication channels including mass media. This could include use of newspapers, radio and television, and targeting facilities such as schools, health facilities, churches and mosques. Although high socio-economic status may promote use of malaria prevention methods such as ITNs [31] due to increased purchasing power, it has not been demonstrated as having a substantial effect on the use of various malaria prevention methods [32] as established in this study. However, ownership of a motor vehicle, which may relate to household income, was found to be associated with use of integrated malaria prevention (AOR 1.75; 95% CI 1.03–2.98). Nevertheless, the socio-economic status index, which was developed using several household facilities and parameters, is a better measure of household wealth [33]. Therefore, increase in socio-economic status in the community alone may not necessarily improve malaria prevention practices especially when barriers at individual or household level do exist.

A limitation of the study is that ownership and use of mosquito nets as one of the methods in the integrated approach could have been under reported as the community was anticipating to receive free LLINs from Government. This implies that some of those who had nets may not have disclosed so in fear of missing out during the Government programme. However, this was minimised by clearly explaining to the participants the purpose of the research before seeking consent hence participation in the study. It was also stressed that personal details such as name of household head that could be used to associate information given to respective households were not required. In this study, utilisation of integrated malaria prevention was defined as use of at least two methods at a household out of the probable twelve. This was because of the low use of other malaria prevention methods besides mosquito nets. However, it is desirable that households use not only two but as many methods as possible and feasible under the circumstances to have a cumulative protective effect against mosquitoes in the integrated approach. Future studies may use a higher cut-off in terms of the number of methods while assessing utilization of integrated malaria prevention. It is also worth noting that some methods in the integrated approach are more effective in preventing malaria than others hence do not have equal protective effect.

## Conclusion

Knowledge on various malaria prevention methods was high and perceptions on use of the integrated approach to malaria prevention were promising. However, there were concerns about the many methods, cost implication and potential health effects of some of the methods. Practices on integrated malaria prevention were low as mosquito nets were predominantly used as a single method. The use of the integrated approach can be improved by promoting use of multiple malaria prevention methods in communities through various communication channels such as mass media.

## Supporting Information

S1 Dataset Study dataset. (SAV)
